# Impact of Sociodemographic Factors on Dental Caries in Children and Availing Fluoride Treatment: A Study Based on National Survey of Children’s Health (NSCH) Data 2016-2019

**DOI:** 10.7759/cureus.18395

**Published:** 2021-09-30

**Authors:** Thevasha Sathiyakumar, Deepa Vasireddy, Sumona Mondal

**Affiliations:** 1 Mathematics, Clarkson University, Potsdam, USA; 2 Pediatrics, Pediatric Group of Acadiana, Lafayette, USA

**Keywords:** fluoride varnish, fluoride, national survey of children's health, race, gender, age, hispanic population, sociodemographic differences, dental cavities, teeth decay

## Abstract

Introduction

Dental caries is a global health issue. It is a largely preventable, multifactorial non-communicable disease. Given the gravity of the situation, in 2014 United States Preventive Services Task Force recommended that the primary care physician apply fluoride varnish from the eruption of the first tooth till the child attains five years of age. Using 2016-2019 National Survey of Children’s Health (NSCH) cross-sectional representative data, the aim of this study was to determine if the child’s age, gender, and race are predictors of the child having decayed teeth or cavities in the past 12 months and if they had availed preventative dental services from the dentist in the past 12 months in the US and if so, did they receive fluoride treatment.

Methods

The prevalence of dental caries and dental treatment among children under each category of sociodemographic risk factors were estimated using 2016-2019 NSCH’s cross-sectional representative data on two survey questions. Then, the statistical significance of the association of the categorical risk factors with the prevalence of dental caries and the association of the categorical risk factors with the prevalence of fluoride dental treatment were tested using two-sample proportion tests and chi-square tests. Further, chi-square residual analysis was employed to better understand the nature of the association and to reveal the degree of contribution to the test statistic from each categorical combination of risk factors.

Results

Prevalence and associative risk of tooth decay in children was the highest in the 6-11 years age group across all three years under study. The 6-11 years age group had the highest prevalence and association of receiving fluoride treatment across all three years. In our study, the prevalence of dental caries in children by race varied according to the year. In 2016-2017 and 2018-2019, it was the Hispanic population with the highest prevalence of tooth decay. In 2017-2018 the highest prevalence was seen in the Non-Hispanic Black (NHB) population. Across all three years, the Hispanic population had the highest associative risk of tooth decay. The Non-Hispanic White (NHW) population had the highest prevalence and association with receiving fluoride treatment across all three years. Male children were found to have the higher prevalence and associative risk of decayed teeth across all three survey years.

Conclusion

Dental caries is a worldwide health burden. However, it can be prevented by different precautionary measures. The results of our study revealed that certain sociodemographic factors such as age, gender, and race of the child make certain groups of the child population more at risk for the development of dental caries; most notable findings were that the male children were significantly associated to have decayed teeth and not availing dental fluoride treatment for which limited information is available in the literature. Additionally, the age groups 1-5 and 6-11 were significantly associated with the prevalence of not receiving dental treatment and the prevalence of dental caries, respectively. More active participation of pediatricians in getting trained for the application of fluoride varnish and helping getting their patients established with dental services per recommendations will help streamline preventative dental care.

## Introduction

Dental caries is a global health issue. It is a largely preventable, multifactorial non-communicable disease. Several pathological factors responsible for dental caries are cariogenic pathogens, fermentable carbohydrates, and a longer time of exposure among others. Saliva, fluoride, plaque control, and carbohydrate intake control serve as protective factors. Oral microbiota form a dental biofilm on the tooth surface that serves as the habitat for cariogenic bacteria [1]. Through pH-driven changes in the oral fluid, fluoride through topical effect helps in remineralization of the tooth enamel and prevents demineralization of it as it stays in the saliva for a certain period of time [2,3]. This helps prevent tooth decay which occurs due to pH changes caused by cariogenic bacteria fermenting carbohydrates. The recommended daily adequate intake of fluoride is the same for males and females from ages birth to 18 years of age and the intake varies per age group. For 0-6 months, 7-12 months, 1-3 years, 4-8 years, 9-13 years, and 13-18 years age groups the daily adequate intake in milligrams (mg) is 0.01, 0.50, 0.70, 1.00, 2.00, and 3.00, respectively [4].

Water fluoridation has been a successful intervention over the years in the prevention of dental caries. Fluoride-containing toothpastes have been another efficient vehicle of delivery of fluoride worldwide [5]. According to a study by Walsh et al. different parts per million fluoride concentration toothpastes had different caries preventive effects [6]. The United States Public Health Service had provided recommendations with updates since 1962 on optimal fluoride concentrations through community water systems to prevent dental caries. The current recommendation being 0.7 milligrams/liter of fluoride [7]. The percentage of the population that receives fluoridated water through community water systems has steadily increased from 37.3% in 1964 to 73% by 2018 [8]. In a study by Dye et al. dental caries was found to be the most chronic and preventable disease in children of age group 6-11 years and in adolescents between 12-19 years [9].

Given the gravity of the situation, in 2014 United States Preventive Services Task Force (USPSTF) recommended that the primary care physician apply fluoride varnish from the eruption of the first tooth till the child attains five years of age. It is to be applied two to four times per year [10,11]. It is a B-level recommendation where there is fair level evidence it improves important health outcomes and benefit outweighs harm [12]. Our objective for this study was to determine using 2016-2019 National Survey of Children's Health’s (NSCH) cross-sectional representative data, if the child’s age, gender, and race are predictors of the child having decayed teeth or cavities in the past 12 months and if they had availed preventative dental services from the dentist in the past 12 months in the US and if so, did they receive fluoride treatment.

## Materials and methods

Data sources

The NSCH is a nationwide survey designed to acquire national and state-level prevalence estimates of the physical and emotional health of children aged 0-17 years. The NSCH is sponsored by the Health Resources and Services Administration and Maternal and Child Health Bureau [13]. The survey is executed as part of the State and Local Area Integrated Telephone Survey by the Centers for Disease Control and Prevention, National Center for Health Statistics. The selection process includes a random data collection of one child under age eighteen from each household where the respondent is an adult in the household who is knowledgeable about the child’s health.

The data were collected nationally by sending mail invites to fill online or paper surveys by parents/guardians of children aged 1-17 years by NSCH. A total of 131,774 subjects’ level topical questionnaires data were collected and pre-processed (Table 1).

**Table 1:** Number of completed child-level topical questionnaires for each year

Year of survey	2019	2018	2017	2016
Number of children completing the questionnaires	29,433	30,530	21,599	50,212

The analysis was conducted on this cross-sectional representative data collected from 2016-2019.

Study measures

The association of the categorical risk factors such as age, gender, and race with the prevalence of dental caries and the prevalence of fluoride dental treatment among children aged 1-17 years were investigated based on the responses given for the following survey questions provided in NSCH data (2016-2017, 2017-2018, 2018-2019).

1. “During the past 12 months, has this child had decayed teeth or cavities, age 1-17 years?”

2. “During the past 12 months, what preventive dental services did this child receive: fluoride treatment, age 1-17 years?”

Statistical analysis

Inter-group differences in abundance were evaluated using Z-proportion tests for dichotomous categorical variables and two-way chi-square (χ2) tests for non-dichotomous categorical variables. The chi-square test, which evaluates the significant association between the mutually exclusive categories of the multiple variables, was employed [14,15]. For the first survey question, the contingency table is constructed with rows having categories of sociodemographic factors as provided in Table 2 and the columns having the categories namely children having decayed teeth (TD) and not having decayed teeth (NTD).

**Table 2:** Sociodemographic variables used in the study NHW: Non-Hispanic White; NHB: Non-Hispanic Black; NHA: Non-Hispanic Asian; NHO: Non-Hispanic other

Study variable	Study categories used
Gender	1. Male 2. Female
Age (yrs)	1. 1-5 2. 6-11 3. 12-17
Race/ethnicity	1. Hispanic 2. NHW 3. NHB 4. NHA 5. NHO

For the second survey question, the rows of the contingency table were categories of each sociodemographic factor and the columns were three categories of dental treatment namely received fluoride treatment (RF), received treatment without fluoride (RNF), and not received treatment (NR), respectively. The null hypothesis (H0) of the chi-square test is that no association exists on the categorical variables in the population and they are independent. Larger values of χ2 test statistics are more contradictory to H0. In a similar way, smaller p-value (< 0.05) is contradictory to H0 where p-value is the null probability that χ2 is at least as large as the observed value.

Further, once an association between categorical variables through the chi-square test is established, residual analysis was utilized to identify the degree of contribution to the test statistic from each categorical combination [16,17]. The standardized residuals which are measures of how significant the cells in the contingency table are to the chi-square value, are calculated. A cell-by-cell comparison of observed frequency (count) and the expected frequency which is based on H0 can help to better understand the nature of the association. The larger the positive residuals of the cell, the higher the count and larger the negative residuals, the fewer the count of the categorical combination relevant to the particular cell than what the hypothesis of independence predicts [18]. Therefore, the larger positive residuals and negative residuals indicate a stronger positive and stronger negative association between the categories of each cell respectively. Statistical analyses and information visualizations were performed using R software (version 4.0.0) [19]. All plots were produced using the packages ‘stats’ and ‘corrplot’.

## Results

Table 3 shows a basic comparison of the children having dental caries and not having dental caries with the sociodemographic risk factors based on the first survey question.

**Table 3:** The two-sample proportion test result (a) and the chi-square test result (b) to show the association between the categorical risk factors such as age, gender, race and the prevalence of dental caries based on 2016-2019 NSCH data for the first survey question “During the past 12 months, has this child had decayed teeth or cavities, age 1-17 years?” NHW: Non-Hispanic White; NHB: Non-Hispanic Black; NHA: Non-Hispanic Asian; NHO: Non-Hispanic other, yrs: years; NSCH: National Survey of Children’s Health.

Variables	Categories	Possess dental caries, Total number-n, Prevalence (%)		χ^2^ value	p-value
		Having teeth-decay n (%)	Not having teeth-decay n (%)		
2016-2017					
Gender	Male	3353 (9.59)	31,612 (90.41)	9.49^(a)^	0.001
	Female	2962 (8.90)	30,308 (91.10)		
Age (yrs)	1-5	1062 (5.97)	16,719 (94.03)	176.80^(b)^	<0.001
	6-11	2874 (13.51)	18,392 (86.49)		
	12-17	2379 (8.15)	26,809 (91.85)		
Race	Hispanic	874 (11.51)	6717 (88.49)	729.92^(b)^	<0.001
	NHW	4045 (8.48)	43,661 (91.52)		
	NHB	447 (11.02)	3609 (88.98)		
	NHA	391 (10.79)	3233 (89.21)		
	NHO	558 (10.61)	61,920 (89.39)		
2017-2018					
Gender	Male	2686 (10.36)	23,252 (89.64)	23.06^(a)^	<0.001
	Female	2181 (9.08)	21,848 (90.92)		
Age (yrs)	1-5	776 (6.03)	12,099 (93.97)	600.33^(b)^	<0.001
	6-11	2252 (14.27)	13,528 (85.73)		
	12-17	1839 (8.63)	19,473 (91.37)		
Race	Hispanic	669 (11.49)	5155 (88.51)	70.98^(b)^	<0.001
	NHW	3108 (9.00)	31,434 (91.00)		
	NHB	373 (11.69)	2818 (88.31)		
	NHA	271 (11.04)	2183 (88.96)		
	NHO	446 (11.27)	3510 (88.73)		
2018-2019					
Gender	Male	3132 (10.39)	27,020 (89.61)	8.95^(a)^	0.001
	Female	2661 (9.64)	24,956 (90.36)		
Age (yrs)	1-5	899 (6.09)	13,870 (93.91)	703.12^(b)^	<0.001
	6-11	2671 (14.58)	15,651 (85.42)		
	12-17	2223 (9.01)	22,455 (90.99)		
Race	Hispanic	837 (12.21)	6020 (87.79)	85.10^(b)^	<0.001
	NHW	3714 (9.28)	36,315 (90.72)		
	NHB	427 (11.53)	3275 (88.47)		
	NHA	328 (11.77)	2459 (88.23)		
	NHO	487 (11.08)	3907 (88.92)		

The following are the notable observations about the prevalence estimation of dental caries among children under categories of each risk factor. The prevalence of dental caries among male children was 9.59%, 10.36%, 10.39% in 2016-2017, 2017-2018, 2018-2019 respectively which is higher compared to the prevalence of dental caries among female children. Among children aged 1-17 years, the prevalence of dental caries was highest for the 6-11 age category in all three years. When considering the race risk factor, the higher prevalence of dental caries varied as Hispanic (11.51%), Non-Hispanic Black (NHB) (11.69%), Hispanic (12.21%) in the respective years 2016-2017, 2017-2018, and 2018-2019. Based on these preliminary results of prevalence estimation, the statistical significance of the differences in the prevalence of dental caries under each category of risk factors was tested.

The risk factor gender was categorized as a dichotomous variable with male and female as two categories. Therefore, the two-sample proportion test was used and the results show significant association (p < 0.001) between the prevalence of dental caries among males and females. For the non-dichotomous variables age and race, the chi-square test was used. The results indicated both age and race contradict (p < 0.001) with H0 and implying significant association with the prevalence of dental caries. Overall, Table 3 results depict that significant statistical associations exist between sociodemographic risk factors and prevalence of dental caries for all three years (2016-2017, 2017-2018, 2018-2019).

**Figure 1:**
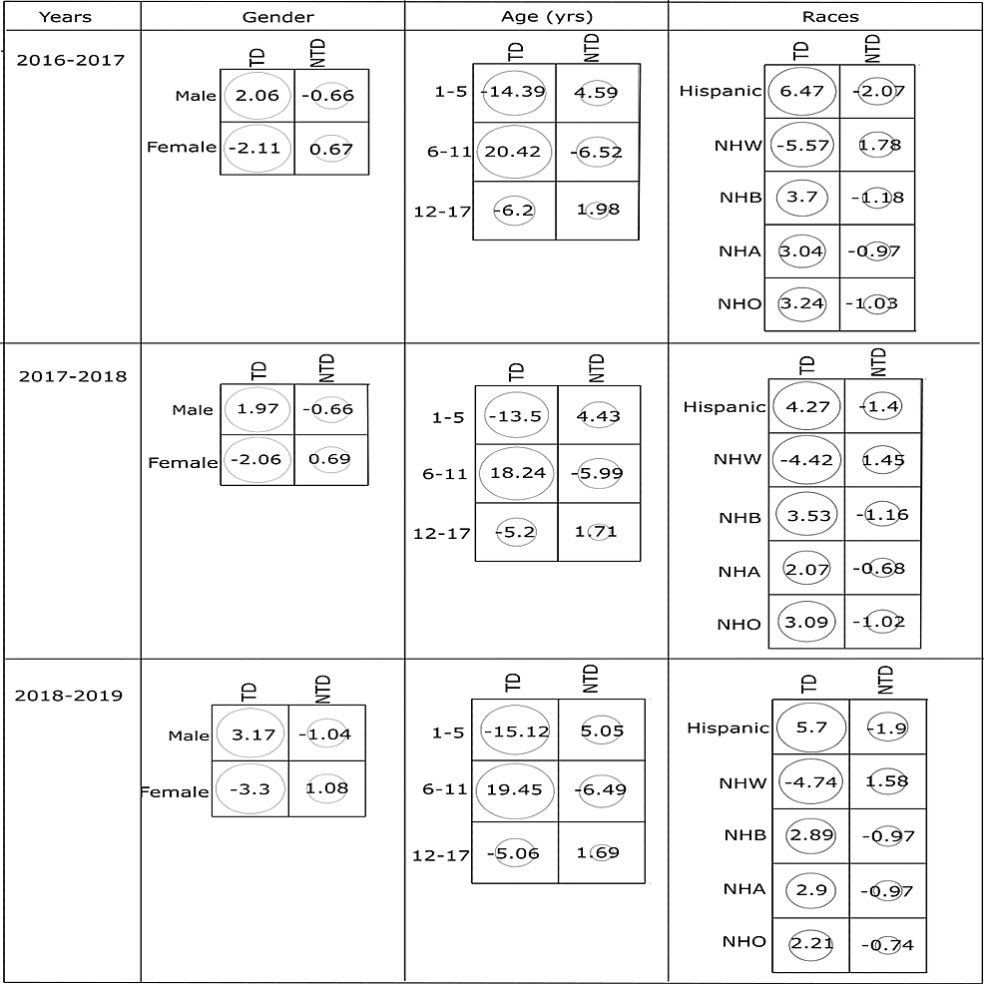
Standardized residuals from χ2 tests for the association between (a) gender, (b) age groups, (c) race on children having decayed teeth (TD) and not having decayed teeth (NTD) during consecutive three-year survey data from 2016-2019. NHW: Non-Hispanic White; NHB: Non-Hispanic Black; NHA: Non-Hispanic Asian; NHO: Non-Hispanic other.

Since the two-sample proportion test and chi-square results show the statistically significant association, the strength of association between the risk factors and prevalence of dental caries was further explored using the standard residuals as shown in Figure 1.

The highest positive value of residuals ($\geq$2) indicates that there is a strong positive association between the group of children having teeth decay with the particular category of each risk factor. Accordingly, it is evident from Figure 1 that the male gender, 6-11 age group, and Hispanic race show a strong positive association with the group of children having teeth decay in all three years.

Next, Table 4 shows a basic comparison of the children who received treatment with fluoride, without fluoride, and not received treatment with the sociodemographic risk factors based on the second survey question.

**Table 4:** The Chi-square test result to show the association between the categorical risk factors such as age, gender, race and prevalence of fluoride dental treatment based on 2016-2019 NSCH data for the second survey question “During the past 12 months, what preventive dental services did this child receive: fluoride treatment, age 1-17 years?” NHW: Non-Hispanic White; NHB: Non-Hispanic Black; NHA: Non-Hispanic Asian; NHO: Non-Hispanic other; yrs: years; NSCH: National Survey of Children’s Health.

Variables	Categories	Dental treatment, Total number-n, Prevalence (%)			χ^2^ value	p-value
		Received fluoride treatment n (%)	Received treatment without fluoride n (%)	Not received treatment n (%)		
2016-2017						
Gender	Male	18,143 (51.84)	10,750 (30.71)	6108 (17.45)	19.17	<0.001
	Female	17,345 (52.18)	10,494 (31.57)	5399 (16.24)		
Age (yrs)	1-5	5480 (30.68)	5032 (28.16)	7352 (41.16)	11056.00	<0.001
	6-11	13,718 (64.54)	5927 (27.88)	1611 (7.58)		
	12-17	16,290 (55.94)	10,285 (35.32)	2544 (8.74)		
Race	Hispanic	3426 (45.23)	2756 (36.38)	1393 (18.39)	713.55	<0.001
	NHW	26,215 (54.87)	13,854 (28.99)	7710 (16.14)		
	NHB	1689 (41.92)	1625 (40.33)	715 (17.75)		
	NHA	1437 (39.91)	1425 (39.57)	739 (20.53)		
	NHO	2721 (51.78)	1584 (30.14)	950 (18.08)		
2017-2018						
Gender	Male	13,200 (50.95)	8126 (31.37)	4580 (17.68)	18.96	<0.001
	Female	12,407 (51.67)	7708 (32.10)	3895 (16.22)		
Age (yrs)	1-5	4150 (32.25)	3636 (28.25)	5081 (39.49)	6799.80	<0.001
	6-11	9972 (63.26)	4496 (28.52)	1296 (8.23)		
	12-17	11,485 (53.96)	7702 (36.18)	2098 (9.86)		
Race	Hispanic	2577 (44.38)	2130 (36.68)	1100 (18.94)	600.14	<0.001
	NHW	18,805 (54.46)	10,228 (29.62)	5500 (15.93)		
	NHB	1241 (39.06)	1320 (41.55)	616 (19.39)		
	NHA	956 (39.21)	948 (38.88)	534 (21.90)		
	NHO	2028 (51.20)	1208 (30.50)	725 (18.30)		
2018-2019						
Gender	Male	15,300 (50.98)	9537 (31.78)	5174 (17.24)	21.71	<0.001
	Female	14,328 (52.11)	8823 (32.09)	4347 (15.81)		
Age (yrs)	1-5	5004 (34.01)	4268 (29.01)	5440 (36.98)	6653.50	<0.001
	6-11	11547 (63.30)	5155 (28.26)	1539 (8.44)		
	12-17	13,077 (53.25)	8937 (36.39)	2542 (10.36)		
Race	Hispanic	3016 (44.33)	2495 (36.67)	1293 (19.00)	787.27	<0.001
	NHW	21,908 (54.92)	11,874 (29.77)	6110 (15.32)		
	NHB	1388 (37.75)	1543 (41.96)	746 (20.29)		
	NHA	1078 (39.02)	1090 (39.45)	595 (21.53)		
	NHO	2238 (51.18)	1358 (31.05)	777 (17.77)		

The preliminary observations based on prevalence estimation are as follows. The prevalence of dental fluoride treatment among female children was 52.18%, 51.67%, 52.11% in 2016-2017, 2017-2018, 2018-2019 respectively which were higher compared to male children. At the same time, the prevalence of untreated caries was higher among male children in all three years. Among children aged 1-17 years, the prevalence of untreated caries was highest for the 1-5 years age category with a decreasing trend in consecutive years. For the risk factor race, Non-Hispanic White (NHW) and Non-Hispanic Asian (NHA) have the higher prevalence of being treated with fluoride and untreated respectively for all three years.

Similar to the first survey question, the statistical significance of the difference in prevalence of treatment under each category of all risk factors was evaluated using chi-square test because the categories of treatment (RF, RNF, NR) considered were non-dichotomous. The chi-square test results indicate all risk factors such as gender, age, and race have a significant association (p < 0.001) with the prevalence of dental fluoride treatment for all three years.

**Figure 2:**
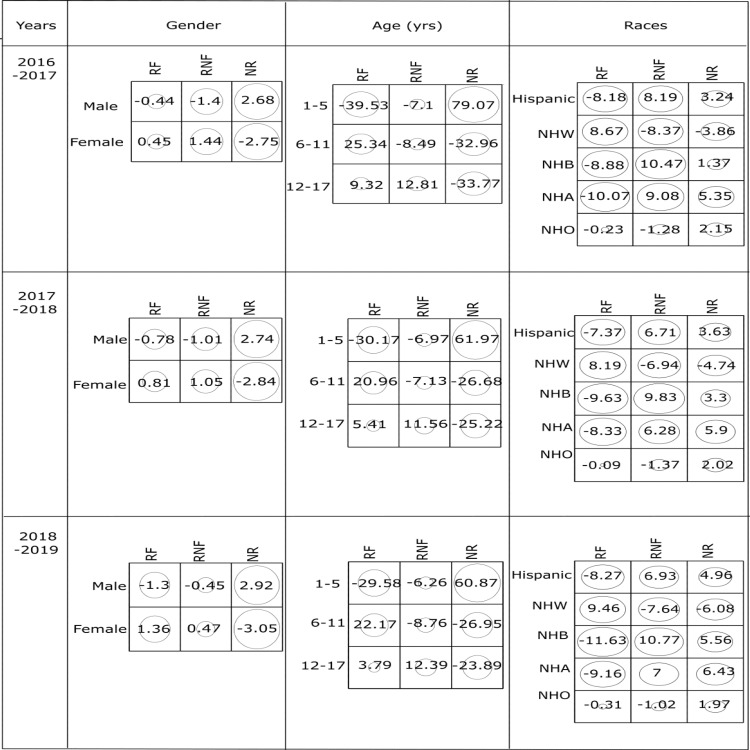
Standardized residuals from χ2 tests for the association between (a) gender, (b) age groups, (c) race on children who received fluoride treatment (RF), received treatment without fluoride (RNF) and not received treatment (NR) during consecutive three-year survey data from 2016-2019. NHW: Non-Hispanic White; NHB: Non-Hispanic Black; NHA: Non-Hispanic Asian; NHO: Non-Hispanic other.

Further, Figure 2 shows that the NR group and the group of male children have a strong positive association with residuals 2.68, 2.74, 2.92 for the years 2016-2017, 2017-2018, and 2018-2019, respectively.

Since the absolute residuals for RF and RNF categories are less than 2, the strength of association is not significant for interpretation as those cells show lack of fit to H0 [16,18]. When age is considered, there is a strong positive association among RF group with the age group 6-11 years, RNF group with the age group 12-17 years, and NR group with age group 1-5 years. When race is considered, there exists a strong positive association between the RF group and NHW race. Both NHB and NHA races have stronger positive associations with the RNF group along with a strong positive association among the NR group with NHA races. Furthermore, the results for strength of association between two categorical variables based on standard residuals for 2017-2018, and 2018-2019 are similar to that of the cohort from 2016-2017.

## Discussion

This study assessed the prevalence of the population with teeth decay and dental care availed across gender, age, and race over a three-year period from 2016 to 2019. We also assessed the associative risk between tooth decay and lack of dental care with the child’s gender, age, and race. The first clinical oral examination is recommended upon the eruption of the first tooth and no later than a year of age [20]. Around 20% of the children between the age group of 5 years to 11 years and around 13% of children aged 12 years to 19 years are found to have at least one untreated decayed tooth [21]. Despite dental visits increasing since 1997 to 2018 across gender and race of children aged 2-17 years, the role of the health care provider recommending the child see a dentist early on in life has scope for improvement [22]. In 2015, 48% of children from birth to 20 years of age had a dental visit which was a 42% increase from children in 1996.

According to the 2015-2016 NCHS data, the highest percentage of total caries was in the age group of 12-19 years and untreated caries was found to be the highest percentage in the 6-11 years age group [23]. Risk factors for early childhood caries (ECC) vary by age. Children going to bed with a bottle of juice, formula or milk or those who consume snacks multiple times a day are at a higher risk of ECC [24]. In our study, the prevalence of teeth decay in children was the highest in the 6-11 years age group across all three years. Across the years 2016-2019 the 6-11 years age group had the highest associative risk with teeth decay. It was noted that the 1-5 years age group was the one with the highest prevalence of children not receiving dental treatment and with the highest associative risk of not receiving dental treatments. This trend of lack of early onset of dental care, one could find interesting as a possible path into the high prevalence of dental caries into the age group of 6-11 years children. The 6-11 years age group had the highest prevalence and association of receiving fluoride treatment across all three years.

Across all three years, the Hispanic population had the highest associative risk of tooth decay. In our study, the prevalence of dental caries in children by race varied according to the year. In 2016-2017 and 2018-2019, it was the Hispanic population with the highest prevalence of tooth decay. In 2017-2018, the highest prevalence was seen in the NHB population. The NHA population has the highest prevalence and associative risk of not receiving dental treatment across all three years. The NHW population had the highest prevalence and association with receiving fluoride treatment across all three years.

According to another study, in 2015 compared to 1996, Hispanics were more likely to have a dental visit which was an increase to 33 percent from 29 percent [25]. In data from prior to National Health and Nutrition Examination Surveys (NHANES), it was found that Hispanic children had the highest prevalence of total caries which includes both treated and untreated caries. The prevalence of untreated caries was the highest in NHB youth [26]. In a study that included children aged 2-17 years, it was noted that ethnic disparities in availing annual dental care persisted in the NHB children across all income levels [27]. Race and ethnicity and the associated cultural behavioral patterns attached with it have resulted in statistically significant association with early childhood caries [28].

Our study is the first to have assessed the prevalence and associative risk the gender of the child carried over the three-year period from 2016 to 2019, for which limited information is available in the literature. Male children were found to have a higher prevalence of decayed teeth across all three survey years. In addition to that, they had a higher associative risk with decayed teeth. The male population had a higher prevalence of those who did not receive dental treatment as well as the greater associative risk of not availing dental treatment.

Unmanaged dental caries leads to major impacts on the child’s health leading to pain, inability to eat and chew, and affects their body weight and growth. Self-esteem and communication are also impacted and declining school attendance and difficulty concentration have been reported [29,30]. Ease of dental referrals seemed to be a hindrance where 55% and 38% reported difficulty in arranging dental referral for uninsured and Medicaid patients, respectively [31]. The child’s behavior affects the dental care that can be provided. According to one study, children who attended daycare showed positive behavior at their dental visit [32]. Fluoride varnish of concentration 5% is used for preventative care. According to a systematic review done in 2017, preventive fractions for the 5% fluoride varnish ranged from 5% to 63% [33]. In our study, female children had a higher prevalence of receiving fluoride treatment and a higher proportion amongst the children for it across all three years. The 6-11 years age group had the highest prevalence and proportion of availing fluoride treatment across all three years. NHW was the most prevalent race for getting fluoride treatment and also had the highest association across all three years. Over time, the American Academy of Pediatrics has been enabling pediatricians and ancillary medical staff in identifying oral disease and providing caries preventive services. According to a recent university-affiliated clinic-based study, improvement in adherence was noted in application of fluoride varnish per guidelines without affecting the flow of patients [34].

Limitations

Due to the nature of the survey response data used in the study, cross-tabulation analysis (chi-square test and standardized residuals) was used to find insights of unnoticed statistically significant associations of the categorical risk factors with both dental caries and fluoride treatment availed by children aged 1-17 years. The authors do acknowledge that the survey data used could have faced recollection bias from survey respondents.

## Conclusions

Dental caries is a global health burden. It is fortunately widely preventable by different precautionary measures. The results of our study revealed hidden insights that certain demographic factors such as age, sex, and race of the child make certain groups of the child population more at risk for the development of dental caries; most notable findings were that the male children were significantly associated to have decayed teeth and not availing dental fluoride treatment for which limited information is available in the literature. Additionally, the age groups 1-5 and 6-11 were significantly associated with the prevalence of not receiving dental treatment and the prevalence of dental caries, respectively. More active participation of pediatricians in getting trained for the application of fluoride varnish and helping getting their patients established with dental services per recommendations will help streamline preventative dental care.
